# Blood-supplementing effect of low molecular weight peptides of E-Jiao on chemotherapy-induced myelosuppression: evaluation of pharmacological activity and identification of bioactive peptides released *in vivo*

**DOI:** 10.3389/fphar.2024.1366407

**Published:** 2024-06-05

**Authors:** Jinju Zhang, Danlin Lin, Yuting Wu, Lixia Chen, Zhiguo Ma, Menghua Wu, Xindan Liu, Ying Zhang, Hui Cao

**Affiliations:** ^1^ Research Center for Traditional Chinese Medicine of Lingnan (Southern China), Jinan University, Guangzhou, China; ^2^ National Engineering Research Center for Modernization of Traditional Chinese Medicine Lingnan Resources Branch, Guangzhou, China; ^3^ Guangdong Province Key Laboratory of Pharmacodynamic Constituents of TCM and New Drugs Research, Guangzhou, China; ^4^ Guangdong Key Laboratory of Traditional Chinese Medicine Information Technology, Guangzhou, China; ^5^ School of Medicine, Foshan University, Foshan, China

**Keywords:** Asini Corri Colla, donkey-hide gelatin, blood deficiency, peptidomics, anemia, network pharmacology

## Abstract

**Background:** *Equus asinus L.* [Equidae; Asini Corri Colla] (donkey-hide gelatin, E-Jiao) is a traditional Chinese medicine renowned for its exceptional blood-supplementing effect. However, the specific components that contribute to its efficacy remain elusive. This study aimed to demonstrate that peptides are responsible for E-Jiao’s blood-supplementing effect and to explore the specific peptides contributing to its efficacy.

**Methods:** The low molecular weight peptides of E-Jiao (LMEJ) were obtained using an *in vitro* digestion method. LMEJ and peptides in the rat bloodstream were characterized by peptidomics analysis. The blood-supplementing effect of LMEJ was assessed using blood-deficient zebrafish and mouse models. The effect of the peptides detected in rat blood was evaluated using the same zebrafish model, and network pharmacology analysis was performed to investigate the underlying mechanisms.

**Results:** A total of 660 unique peptides were identified within LMEJ. Both E-Jiao and LMEJ significantly alleviated myelosuppression in mice but only LMEJ attenuated myelosuppression in zebrafish. After the administration of E-Jiao to rats, 67 E-Jiao-derived peptides were detected in the bloodstream, 41 of which were identical to those identified in LMEJ. Out of these 41 peptides, five were synthesized. Subsequent verification of their effects revealed that two of them were able to alleviate myelosuppression in zebrafish. Network pharmacology study suggested that E-Jiao may exert a blood-supplementing effect by regulating signaling pathways such as JAK-STAT, IL-17 and others. These results indicated that peptides are at least partially responsible for E-Jiao’s efficacy.

**Conclusion:** This study provides a crucial foundation for further exploration of the bioactive components of E-Jiao.

## 1 Introduction

Cancer is a significant threat to human lives due to its high incidence and mortality rates. While new cancer therapies have been developed, radiotherapy and chemotherapy remain the primary approaches used. Unfortunately, these treatments often cause severe myelosuppression, which can lead to reductions in various types of blood cells (Boccia et al., 2022). Chinese medicine considers this myelosuppression to be one of the blood-deficient syndromes, which commonly includes impaired hematopoietic function, decreased blood cells and hemoglobin, and weakened immune function (Li et al., 2016). E-Jiao, also known as Colla Corii Asini and donkey-hide gelatin, is a solid glue made by extracting and concentrating collagens from donkey (Equidae; *Equus asinus* L.) hides (Committee Chinese Pharmacopoeia, 2020). E-Jiao has been recognized as a tonic medicine with blood-supplementing effect and is commonly used to treat blood deficiency. Modern research and clinical applications have demonstrated its effectiveness in treating myelosuppression caused by chemotherapy or radiotherapy (Li et al., 2016; Zhang et al., 2021; Wu et al., 2022). However, to our knowledge, the specific components responsible for the blood-supplementing effect of E-Jiao remain unclear.

Proteins, especially type I collagen, are the main components of E-Jiao, constituting for 80% of its total composition (Wang et al., 2014). It has been postulated that ingested proteins need to be digested into low molecular weight peptides or amino acids before they can be absorbed and exert their beneficial effects (Miner-Williams et al., 2014). Peptides have long been recognized as crucial for regulating various physiological processes, such as signaling, homeostasis, immune regulation and hematopoiesis (Petrov et al., 1997; Kisling et al., 2019; Yu et al., 2020). Peptides derived from foods often share structural similarities with endogenous peptides, suggesting that they have the potential to exert a variety of activities (Cunningham et al., 2017; Sato, 2018). Several studies have utilized *in vitro* digestion methods to produce E-Jiao peptides and have shown that these peptides have various activities, such as antioxidant, anti-inflammatory, immunomodulatory and antianemia effects (Xiao et al., 2020; Cheng et al., 2021; Xiao et al., 2022; Cheng et al., 2023; Yu et al., 2023). An *in vitro* activity-guided fractionation study also revealed that a peptide derived from E-Jiao exhibits hematopoietic effect in mice (Wu et al., 2016). These results suggest that peptides may, at least in part, be responsible for the blood-supplementing effect of E-Jiao.

The genetic program that regulates the development and differentiation of hematopoietic cells is highly conserved between zebrafish and mammals, making zebrafish a powerful vertebrate model for studying hematopoietic diseases (Kulkeaw and Sugiyama, 2012). Moreover, a significant number of peptides are produced *in vivo* through the enzymatic digestion of E-Jiao. However, correlating the *in vitro* activity of peptides with their *in vivo* activity may be challenging because peptides need be absorbed into the bloodstream to exert their effects (Miner-Williams et al., 2014). Therefore, it is crucial to investigate E-Jiao-derived peptides that enter the bloodstream to better understand the bioactive components of E-Jiao. Recently, peptidomics has become a powerful tool with which E-Jiao-derived peptides can be directly identified in the bloodstream (Sheng et al., 2021).

In this study, we hypothesized that low molecular weight peptides are responsible for the blood-supplementing effect of E-Jiao. We prepared low molecular weight peptides from E-Jiao (LMEJ) using an *in vitro* digestion method and characterized LMEJ through peptidomic analysis. Subsequently, the blood-supplementing effect of E-Jiao and LMEJ was evaluated using a zebrafish model and a mouse model. The profile of E-Jiao-derived peptides in rat blood after oral administration of E-Jiao was thoroughly investigated and compared to LMEJ. Then, E-Jiao-derived peptides in rat blood were selected, and their effect was evaluated using the same zebrafish model. Network pharmacology was used to explore the possible mechanisms. This research may provide new insights into the bioactive components responsible for E-Jiao’s blood-supplementing effect.

## 2 Materials and methods

### 2.1 Chemicals and reagents

Commercial E-Jiao was obtained from Gansu Tianshui Xihuang E-Jiao Co., Ltd. (Batch No. 17101022, SFDA Approval Number: Z62021196, Tianshui, Gansu, China). The E-Jiao was prepared using donkey hides obtained from domesticated donkeys and was in accordance with the quality standard of the Chinese Pharmacopoeia (2020 edition) (Committee Chinese Pharmacopoeia, 2020). Commercial Danggui Buxue Koufuye was purchased from Zhengzhou Union Pharmaceutical Factory (Batch No. 012303161, SFDA Approval Number: Z10970001, Zhengzhou, Henan, China). The Danggui Buxue Koufuye is a liquid preparation obtained by extracting Astragali Radix (*Astragalus membranaceus* (Fisch.) Bge.var.*mongholicus* (Bge.) Hsiao or *Astragalus* membranaceus (Fisch.) Bge.) and Angelicae Sinensis Radix (*Angelica sinensis* (Oliv.) Diels) according to the method of Chinese Pharmacopoeia (2020 edition) and meets the quality criteria specified in the Chinese Pharmacopoeia (2020 edition) (Committee Chinese Pharmacopoeia, 2020). Commercial Leucogen tablets were supplied by Jebel Pharmaceuticals (Batch No. 221110, SFDA Approval Number: H32025444, Zhenjiang, Jiangsu, China). Porcine pepsin (1:15,000) and porcine trypsin (≥2500 units/mg) were purchased from Aladdin Chemistry Co., Ltd. (Shanghai, China). Vinorelbine tartrate, cyclophosphamide and Sudan Black B were purchased from Macklin, Inc. (Shanghai, China). Doxorubicin was obtained from Meilunbio Biochemical Co., Ltd. (Dalian, China), while *o*-dianisidine was purchased from TCL (Shanghai, China). Peptides (≥98% pure) were synthesized by China Peptides (Shanghai, China). MS-grade formic acid and acetonitrile were purchased from Thermo Fisher Scientific (Waltham, MA, United States). Ultrapure deionized water was obtained from a Milli-Q water purification system (MA, United States).

### 2.2 *In vitro* preparation and characterization of the peptides from E-Jiao

#### 2.2.1 *In vitro* gastrointestinal digestion

The *in vitro* simulated gastroduodenal digestion of E-Jiao was performed according to a previous method, with slight modifications (Minekus et al., 2014). E-Jiao was dissolved in deionized water at a concentration of 50 mg/mL, and the resulting solution was centrifuged for 5 min at 10,000 × g at 4°C. For the gastric phase, the pH of the E-Jiao solution was adjusted to 2.0 using 1 mol/L HCl, and subsequently, pepsin was added at an enzyme-to-substrate ratio of 1:250 (w/w). The mixture was incubated at 37°C in a shaking incubator for 3 h. The gastric phase was terminated by adjusting the pH to 7.0 with 1 mol/L NaOH. Moving to the duodenal phase, the solution was incubated with trypsin at an enzyme-to-substrate ratio of 1:250 (w/w) at 37°C for 2 h. At the end of the duodenal phase, trypsin was inactivated by heating the digests at 50°C for 10 min. Subsequently, the digests were centrifuged at 10,000 × g for 10 min. The fraction of the digest with a molecular weight less than 10 kDa, representing LMEJ, was obtained by successively centrifuging the supernatants using an Amicon Ultra 15 mL 30 K centrifugal filter (Millipore, Gloucestershire, United Kingdom) and an Amicon Ultra 15 mL 10 K centrifugal filter (Millipore, Gloucestershire, UK), both at 3500 × g for 30 min. The resulting ultrafiltrate was desalted by dialysis using a membrane with a 100 Da molecular weight cutoff (Yuan Ye Bio-Technology Co., Ltd., Shanghai, China). Subsequently, the ultrafiltrate was lyophilized and stored at −20°C. The entire *in vitro* digestion experiment was performed independently four times.

#### 2.2.2 Peptide analysis

LMEJ was characterized by liquid chromatography–tandem mass spectrometry (LC‒MS/MS) analysis, which was carried out on a Q Exactive Plus Orbitrap mass spectrometer equipped with an EASY-nLC 1200 HPLC system (Thermo Fisher Scientific, Waltham, MA, United States). Before analysis, the samples were dissolved in 0.1% formic acid in water at a concentration of 1 μg/μL, loaded onto a trap column (Acclaim PepMap 100 C18, 5 μm, 100 μm × 20 mm; Thermo Fisher Scientific, San Jose, CA, United States) and subsequently separated with an analytical column (Acclaim PepMap 100 C18, 2 μm, 50 μm × 150 mm). The mobile phase consisted of two solutions: eluent A was MS-grade water with 0.1% formic acid (v/v), and eluent B was acetonitrile with 0.1% formic acid (v/v). The flow rate was set at 300 nL/min. The gradient profile was as follows: 0–51 min, 4%–28% B; 51–56 min, 28%–38% B; 56–60 min, 38%–90% B; and 60–70 min, 90% B. The MS settings were as follows: spray voltage of 2.2 kV in positive mode. For full MS data acquisition, the resolution was set to 70,000 with a scan range of 350–1500 m/z, the AGC target was set to 1 × 10^6^, and the maximum IT was 50 ms. For dd-MS^2^, the AGC target was 5 × 10^6^, and the maximum IT was 120 ms. The 10 most intense precursors were selected for fragmentation with a resolution of 17,500 and a dynamic exclusion of 12 s. Ions with 1, 7, 8 and 9 charges were excluded from MS/MS selection.

The raw LC–MS/MS data files were analyzed using Proteome Discoverer software (version 2.2; Thermo Fisher Scientific, United States). Due to the incompleteness of the UniProt *E. asinus* database, the UniProt *Equus caballus* database was also used for the search. Considering the enzymes used in digestion, reference sequences of porcine pepsin (Entry number: P00791) and porcine trypsin (Entry number: P00761) were also included in the database search to exclude peptides originating from these enzymes. The search parameters were set as follows: mass analyzer, Orbitrap mass spectrometer; enzyme, not specific; minimum peptide length, 6; precursor mass tolerance, 10 ppm; fragment mass tolerance, 0.02 Da; and dynamic modification, oxidation/+15.995 Da (M); additionally, no static modification was applied. The significance threshold was set at *p* < 0.05. The data was filtered using Percolator with a false discovery rate of ≤0.01.

### 2.3 Zebrafish experiments

#### 2.3.1 Zebrafish maintenance

Wild-type adult zebrafish and transgenic Tg (*lyz:DsRED2*) adult zebrafish, which express red fluorescent protein in their neutrophils, were obtained from the China Zebrafish Resource Center (Wuhan, China) and raised in the zebrafish facility at the College of Pharmacy of Jinan University. All experiments were conducted following the guidelines of the Jinan University Animal Care and Use Committee. Embryos obtained from paired mating were collected and maintained in egg water at a temperature of 28.5°C as described in the Zebrafish Book (Westerfield, 2000).

#### 2.3.2 Effect of LMEJ and E-Jiao on zebrafish

At 24-hour-postfertilization (hpf), 20 wild-type zebrafish embryos were randomly distributed into each well of 24-well plates after the egg membrane was removed using 0.5 mg/mL chain protease. The groups were assigned as follows: blank control (Control), model (Model), positive controls (Danggui Buxue Koufuye (DGBX) and leucogen tablet (LXS)), E-Jiao (E-Jiao), low-dose LMEJ (LMEJ-L), medium-dose LMEJ (LMEJ-M) and high-dose LMEJ (LMEJ-H). The Control group was treated with embryo medium, while the Model group was treated with 10 μg/mL doxorubicin in embryo medium. The DGBX, LXS, E-Jiao, LMEJ-L, LMEJ-M and LMEJ-H groups were treated with 10 μg/mL doxorubicin combined with 4.5 mL/L Danggui Buxue Koufuye, 15 μg/mL leucogen, 100 μg/mL E-Jiao, 25 μg/mL LMEJ, 50 μg/mL LMEJ and 100 μg/mL LMEJ, respectively. Each group had three replicates. The embryos were exposed for 24 h. To avoid melanin interference, the embryos were treated with 0.045 g/L 1-phenyl-2-thiourea at 12 hpf to inhibit melanin synthesis. The erythrocyte counts in the zebrafish embryos were evaluated with *o*-dianisidine staining performed according to previous methods (Yang et al., 2015). After staining, the embryos were photographed using a biomicroscope (Olympus, Japan). The erythrocyte count in the yolk sac of zebrafish larvae was quantified based on the staining intensity of the erythrocytes using ImageJ (version 5.1). At 48 hpf, Tg (*lyz:DsRED2*) zebrafish embryos were grouped in the same way as wild-type zebrafish embryos but treated with 25 μg/mL vinorelbine tartrate instead of doxorubicin. After 24 h of exposure, images of each Tg (*lyz:DsRED2*) zebrafish embryo were acquired and collected using an MZ10F fluorescence stereomicroscope (Leica, Germany). The neutrophils in the cloaca-to-tail region of zebrafish were also quantified using ImageJ (version 5.1).

### 2.4 Mouse experiments

#### 2.4.1 Animals and treatments

Sixty-four specific pathogen-free (SPF) Balb/c mice (6 weeks old weighing 16–18 g) were obtained from Charles River (Beijing, China). The mice were kept under standard conditions (temperature, 22°C ± 2°C; humidity, 55% ± 5%) at the Laboratory Animal Center of South China University of Technology (SCUT). All animal procedures were conducted following the Guidelines for the Care and Use of Laboratory Animals of SCUT and were approved by the Laboratory Animal Ethics Committee of SCUT (approval number: 2023094).

The mice were randomly assigned to eight groups, which were the same as those described for the zebrafish experiments. To establish a myelosuppressed mouse model, the mice in all groups except the Control group were intraperitoneally injected with 100 mg/kg body weight cyclophosphamide once a day on days 1, 2, 3, and 6. According to the ratio of the clinical daily dose in humans to the human-mouse body surface area ratio, the daily intragastric doses of Danggui Buxue Koufuye (20 mL/d for humans) and leucogen tablets (60 mg/d for humans) were determined to be 2.6 mL/kg and 7.8 mg/kg, respectively. The mice in the E-Jiao group were orally administered a solution of E-Jiao (6 g/d for humans) at a dose of 1.56 g/kg, which is equivalent to double the clinical application dose. Considering that LMEJ was obtained in a 50% yield after *in vitro* simulated gastroduodenal digestion of E-Jiao, the mice in the LMEJ-L group received a daily oral dosage of 0.39 g/kg LMEJ, which is equivalent to the clinical application dose. The LMEJ-M and LMEJ-H groups received LMEJ at dosages of 0.78 g/kg daily and 1.56 g/kg daily, respectively. The Control and Model groups were orally administered the same volume of ultrapure water daily. The body weights of the mice were recorded daily until the termination of the experiment.

#### 2.4.2 Visceral indices and routine blood test

The mice were anesthetized via isoflurane inhalation and blood was collected via the eyeball removal method on day 11. After blood collection, the mice were immediately euthanized by cervical dislocation. The thymus and spleen were promptly excised, rinsed with water, dried on filter paper and weighed. Each organ index was calculated as the weight of the spleen (or thymus) relative to the mouse’s body weight (g/kg). Whole blood was collected into a 1.5 mL tube containing EDTA-2Na (Solaibao Biological Technology Co., Ltd., Beijing, China) for hematological tests, which included white blood cell (WBC), neutrophil (NE), lymphocyte (LY), monocyte (MO), eosinophil (EO), basophil (BA), red blood cell (RBC) and platelet (PLT) counts. These tests were carried out using a Hemavet 950 blood analyzer (Drew Scientific Group, United Kingdom) following the manufacturer’s instructions. All blood samples were analyzed within 2 hours after collection.

### 2.5 Rat experiments

#### 2.5.1 Animals and treatments

Ten eight-week-old male Wistar rats (SPF, Charles River, Guangzhou, China; 280–320 g) were purchased from Charles River (Beijing, China) and housed at the Laboratory Animal Center of SCUT under controlled conditions (temperature, 22°C ± 1°C; humidity, 55% ± 5%). After acclimatization for 1 week, the rats were fasted for 24 h before receiving E-Jiao. E-Jiao was dissolved in ultrapure water and administered orally to each rat at a dose of 0.7 g/kg. Blood samples were collected as previously described with minor modifications (Sheng et al., 2021). The rat was anesthetized with isoflurane and approximately 300 μL of blood was collected from the jugular vein before drug administration and at 1 h and 3 h after administration. Blood was collected in a 1.5 mL tube containing EDTA-2Na (Solaibao Biological Technology Co., Ltd., Beijing, China) and immediately centrifuged at 3000 *g* for 15 min at 4°C to obtain plasma. The collected plasma was stored at −80°C until further use. The entire experiment was repeated three times, with intervals of at least 7 days to allow the rats to recover. All procedures involving animals were approved by the Laboratory Animal Ethics Committee of South China University of Technology (approval number: 2023027). At the end of each experiment, four individual plasma samples were selected for each time point and used for subsequent peptide extraction.

#### 2.5.2 Extraction of peptides for MS analysis

Plasma sample preparation for MS analysis was performed according to previous methods, with minor modifications (Sheng et al., 2021). Specially, 100 μL of each plasma sample was mixed with four volumes of extraction buffer containing 20% acetonitrile, 0.1% formic acid and 0.1% sodium chloride. The mixture was subsequently transferred to an Amicon Ultra 0.5 mL 3 K centrifugal filter (Millipore, Gloucestershire, United Kindom) and centrifuged at 14,000 × g for 30 min at 4°C. The filtrate was mixed with 100 μL of 0.1% formic acid and then desalted using a Waters Sep-Pak C18 column (Waters, Massachusetts, United States) following the manufacturer’s guidelines. The eluted fraction was freeze-dried and stored at −80°C before LC–MS/MS analysis.

#### 2.5.3 Peptide identification

The same LC–MS/MS method described in Section 2.2.2 was used to identify E-Jiao-derived peptides in blood samples, with some modifications made to the database searching. The raw data was searched against a homemade database created according to a previous study (Zhang et al., 2023) and the Swiss-Prot database of *Rattus norvegicus* (accessed in February 2023, 10,128 entries) using the parameters described in Section 2.2.2. The homemade database consisted of the 100 most abundant E-Jiao proteins, including type I collagen, type III collagen and others. The identified peptides were manually verified to ensure that each peptide was detected in at least two samples at each time point. Considering that a database search was not conducted for the entire rat proteome, E-Jiao-derived peptides were verified by searching against UniProtKB/Swiss-Prot (Swiss-Prot) using the BLASTP algorithm (https://blast.ncbi.nlm.nih.gov/Blast.cgi?PROGRAM=blastp&PAGE_TYPE=BlastSearch&LINK_LOC=blasthome) to ensure their uniqueness to E-Jiao-derived proteins. The identified peptides that matched both the E-Jiao-derived and rat-derived proteins were further refined manually to exclude those detected in any samples collected before E-Jiao administration.

### 2.6 Selection of peptides for synthesis and evaluation of their blood-supplementing effect

E-Jiao-derived peptides that were identified in both rat blood and LMEJ were scored by PeptideRanker (http://distilldeep.ucd.ie/PeptideRanker/). Only peptides with a length of less than 20 amino acids, no missed cleavage sites for trypsin and PeptideRanker scores exceeding 0.7 were considered as bioactive peptide candidates. Subsequently, the toxicity of these peptides was assessed by the ToxIBTL (https://server.wei-group.net/ToxIBTL). The novelty of the peptides was checked by searching the BIOPEP database (https://biochemia.uwm.edu.pl/). Five peptides (PP-1, PP-2, PP-3, PP-4 and PP-5) that satisfied the aforementioned criteria and obtained the highest PeptideRanker scores were chemically synthesized. Wild-type zebrafish embryos were used to evaluate the blood-supplementing effect of these peptides, following the procedures as described in Section 2.3.2, with modifications to the grouping and the quantification methods for neutrophils. The Control, Model, DGBX and LXS groups were treated in the same way as described in Section 2.3.2, while the LMEJ group was treated with 100 μg/mL LMEJ. To determine the administration dose, the PP-1∼PP-5 were firstly administrated to 24 hpf zebrafish embryos. The maximum tolerated concentration of each PP was determined based on mortality and malformation observed 48 h postexposure. The maximum dosing concentration of each PP was chosen according to the maximum tolerated concentration observed in zebrafish embryos. Three dose groups of PP were set as follows: low (10 μg/mL for PP-1-L; 3.75 μg/mL for PP-2-L; and 2.5 μg/mL for PP-3-L, PP-4-L and PP-5-L); medium (20 μg/mL for PP-1-M; 7.5 μg/mL for PP-2-M; and 5.0 μg/mL for PP-3-M, PP-4-M and PP-5-M); and high (40 μg/mL for PP-1-H; 15.0 μg/mL for PP-2-H; and 10.0 μg/mL for PP-3-H, PP-4-H and PP-5-H). Erythrocytes from zebrafish were stained and observed according to the procedure as described in Section 2.3.2. To evaluate the changes in neutrophil counts, zebrafish were subjected to the Sudan Black B staining, which was performed as previously described (Chen et al., 2022). All experiments were independently repeated at least three times.

### 2.7 Network pharmacology and molecular docking

#### 2.7.1 Target screening for diseases and peptides

The mechanisms behind the blood-supplementing effect of E-Jiao-derived peptides were investigated using network pharmacology. The SMILES (Simplified Molecular Input Line Entry System) of peptides were acquired via the PepSMI tool (https://www.novoprolabs.com/tools/convert-peptide-to-smiles-string). These SMILES strings were then subjected to target prediction using the Super PRED database (https://prediction.charite.de/). Disease (blood deficiency) related targets were identified by searching the GeneCards (https://www.genecards.org/) and OMIM-GENE-MAP databases (https://omim.org/search/advanced/geneMap) with keywords including “anemia,” “bone marrow suppression,” and “neutropenia”. The disease-related targets obtained from GeneCards were screened three times. Only those with relevance scores above the median were chosen. Targets from both GeneCards and OMIM-GENE-MAP were combined, and duplicates were removed. Finally, gene targets at the intersection of peptides and diseases were determined using Venny 2.1 (http://bioinfogp.cnb.csic.es/tools/venny/index.html).

#### 2.7.2 Construction of the PPI network

The targets that intersected were inputted into the STRING online software (https://cn.string-db.org/), with the research species restricted to “*Homo sapiens*”. A confidence level of 0.700 was set. Subsequently, a network diagram was generated using the Cytoscape 3.10.1 software, and the key targets were identified using the CentiScape tool, based on betweenness centrality, closeness centrality, and degree centrality (Scardoni et al., 2009).

#### 2.7.3 GO and KEGG pathway enrichment analysis

To elucidate the functions and pathways associated with the peptides’ effect on blood deficiency, the key targets were uploaded to Metascape (https://metascape.org/gp/index.html#/main/step1) for enrichment analyses. The enrichment analyses included Gene Ontology (GO) analyses, which cover Biological Process (BP), Cellular Component (CC), and Molecular Function (MF), as well as Kyoto Encyclopedia of Genes and Genomes (KEGG) analyses.

#### 2.7.4 Molecular docking

The molecular docking was carried out to confirm the interactions between peptides and their targets. The three core targets with the highest betweenness centrality were selected for docking with two peptides. The 2D structures of the peptides were transformed into 3D structures using ChemDraw 19.0. Subsequently, AutoDockTools 1.5.6 was utilized to eliminate water molecules, add polar hydrogen atoms, and define rotatable bonds. The crystal structures of the core targets were retrieved from the RSCB PDB database (https://www.rcsb.org). The crystal structures of STAT3 (PDB ID: 6NJS), HSP90AA1 (PDB ID: 3WQ9), and NFE2L2 (PDB ID: 7K2A) were chosen for further analysis. Prior to docking, water molecules and ligands in the crystal structure were removed, and polar hydrogen atoms were added. Docking was then performed using AutoDock Vina 1.1.2, and the results were visualized using PyMOL 2.5.7.

### 2.8 Data analysis

Statistical analysis was conducted using GraphPad Prism (version 8.0.2), utilizing one-way ANOVA, the Kruskal‒Wallis test, or the t‒test to assess intergroup or intragroup differences, respectively. A *p*-value <0.05 was considered to indicate statistical significance. The data are presented as the mean ± standard deviation (SD).

## 3 Results

### 3.1 Peptide profile of LMEJ

**FIGURE 1:**
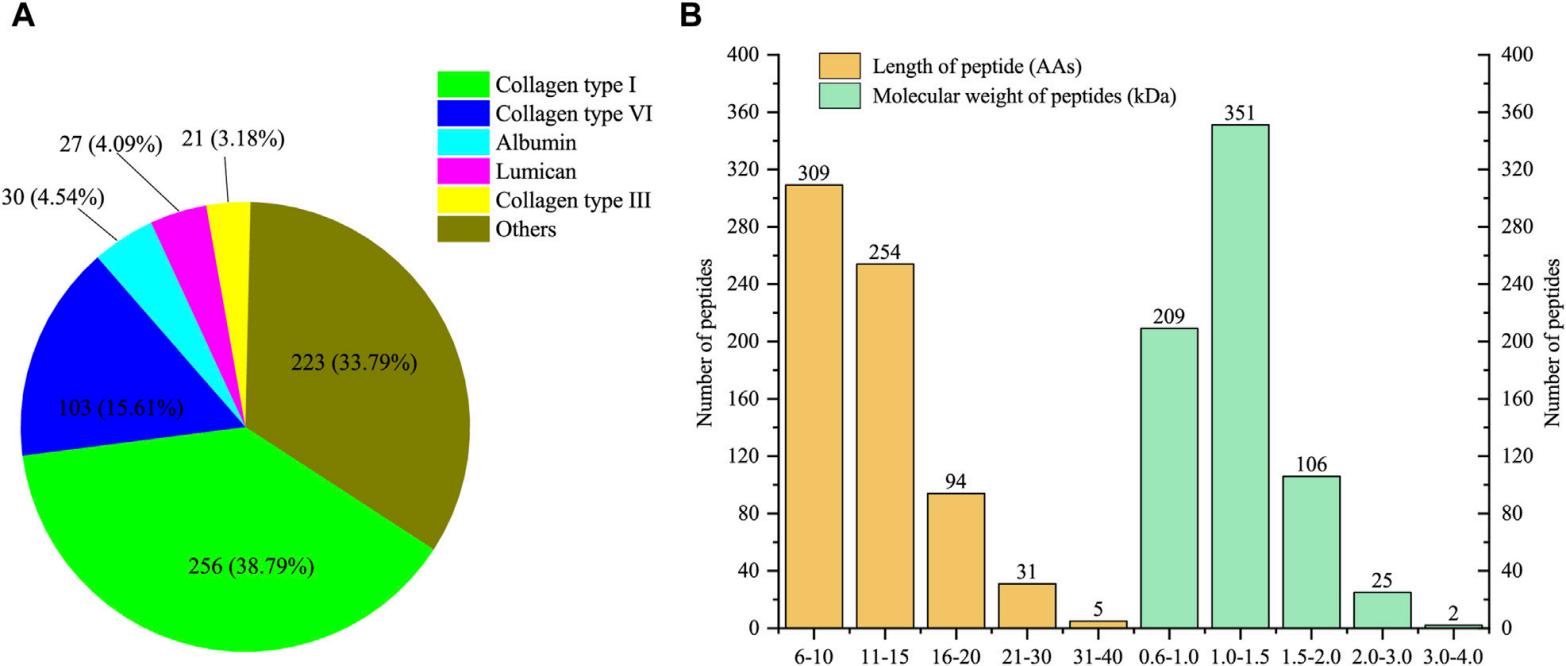
Characterization of low molecular weight peptides from E-Jiao (LMEJ). **(A)** The five proteins with the highest number of unique peptides. **(B)** The distribution of peptide length and molecular weight of all identified peptides.

A total of 693 peptides derived from 58 proteins (partially identical) were identified in four replicates. Among them, 660 unique peptides exclusively matched to one protein. Detailed information about the identified peptides and their corresponding parent proteins can be found in Supplementary Table S1. As shown in Figure 1A, the five proteins associated with the most identified peptides were collagen type I, collagen type VI, albumin, lumican and collagen type III. Notably, collagen type I accounted for 256 unique peptides, comprising 38.79% of the total unique peptides. Collagen type III and collagen type VI also released 21 and 103 unique peptides, respectively. The peptides released from these three types of collagens accounted for 57.58% of the total identified peptides. In addition to collagens, albumin and lumican, released 30 and 27 peptides, respectively. The lengths of all the identified peptides ranged from 6 to 40 amino acids (Figure 1B). Peptides with lengths of less than 15 amino acids composed the majority (81.24%) of the identified peptides. Similarly, the molecular weight distribution exhibited a parallel pattern to the length distribution, spanning from 0.6 to 4.0 kDa. The majority of the peptides had molecular weights ranging from 1.0 to 1.5 kDa, constituting 50.65% of all peptides.

### 3.2 LMEJ alleviated doxorubicin-induced myelosuppression in zebrafish

**FIGURE 2:**
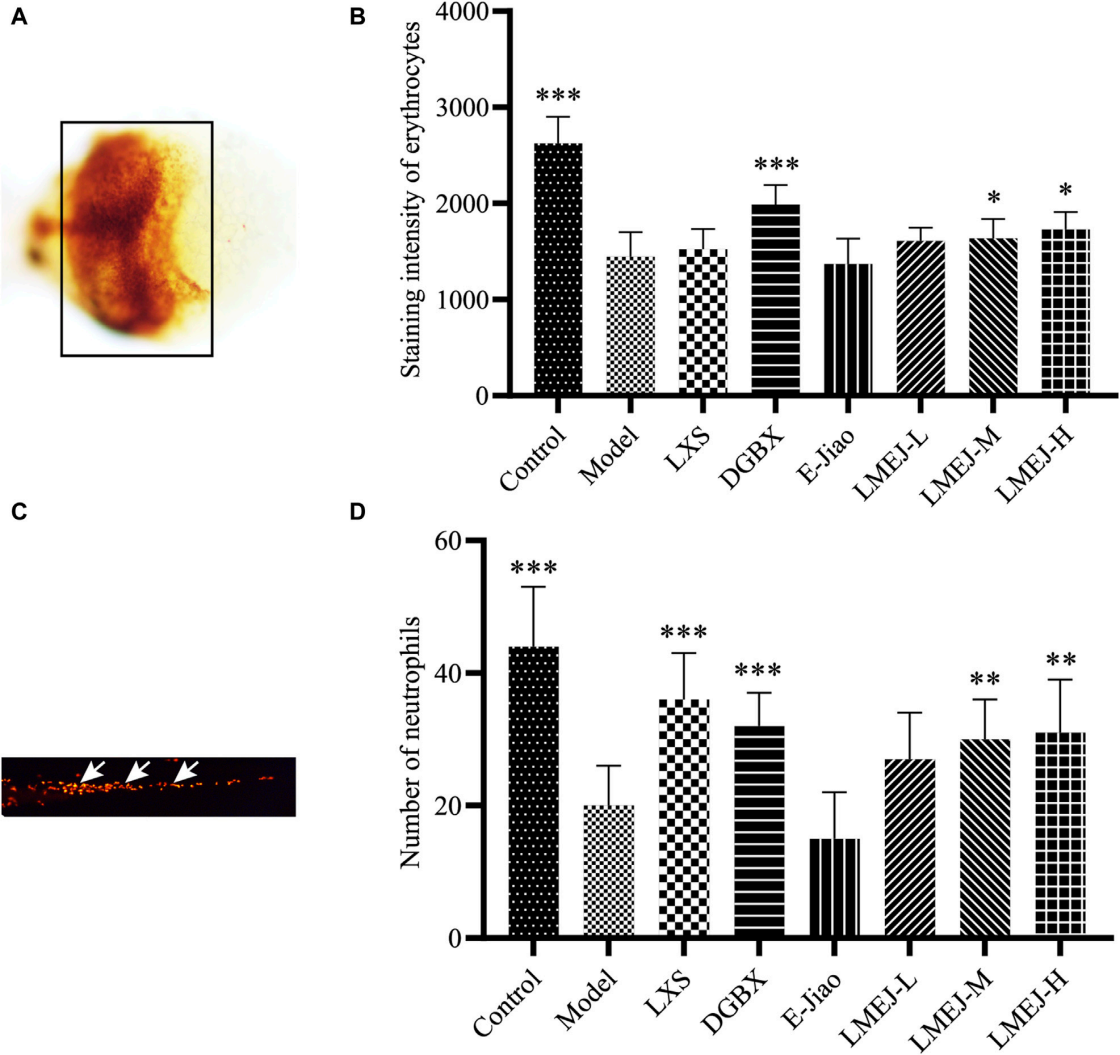
Effect of LMEJ on erythrocytes in doxorubicin-induced myelosuppressed zebrafish and neutrophils in vinorelbine-induced myelosuppressed zebrafish. **(A)** Images of erythrocytes from zebrafish. **(B)** LMEJ protects zebrafish from the doxorubicin-induced decrease in erythrocyte count. **(C)** Images of neutrophils from zebrafish. **(D)** LMEJ alleviated the vinorelbine-induced reduction in neutrophil count in zebrafish. Erythrocytes are indicated by a black box, and neutrophils are indicated by white arrows. The values are expressed as the mean ± SD (n ≥ 60). *p < 0.05, **p < 0.01, ***p < 0.001 vs. the Model group.

Exposure to doxorubicin and vinorelbine led to significant decreases in erythrocyte and neutrophil counts within the zebrafish Model group compared to those in the Control group (Figure 2). These findings indicated a significant inhibition of erythrocytes and neutrophils. The administration of DGBX significantly elevated neutrophil and erythrocyte counts, while LXS significantly elevated neutrophil counts only. Notably, LMEJ exhibited a dose-dependent protective effect on erythrocytes and neutrophils similar to the effects of DGBX and LXS. At a concentration of 100 μg/mL, LMEJ facilitated the recovery of the erythrocyte count compared to the Control group (Figure 2B). A significant difference (*p* < 0.05) was observed between the LMEJ group and the Model group. A similar trend was observed for neutrophil counts, suggesting the therapeutic effect of LMEJ in alleviating the vinorelbine-induced inhibition of neutrophil production (Figure 2D). Interestingly, E-Jiao did not demonstrate a protective effect on either erythrocyte or neutrophil damage in zebrafish (Figures 2B,D).

### 3.3 Effects of LMEJ on the body weight and visceral indices of mice

Intraperitoneal injection of cyclophosphamide for three consecutive days led to a significant reduction in body weight from day 2 to day 5 within the Model group (*p* < 0.01 or *p* < 0.001) compared to the Control group (Supplementary Figure S1A). However, the body weights of the mice in the LMEJ-L group decreased more slowly than other groups. A significant difference (*p* < 0.05) was observed between the LMEJ-L and Control groups on day 5. On day 6, the body weights of the mice in the Model group exhibited slight recovery but decreased again after an additional injection of cyclophosphamide. The mice in the Model group regained weight by day 10, but the values remained significantly lower than the Control group. Moreover, the body weights of the mice in the LMEJ-L and LMEJ-H groups, which were lower than the Control group, were significantly greater than the Model group (*p* < 0.05 or *p* < 0.01) on day 9 and day 10.

Furthermore, intraperitoneal injection of cyclophosphamide caused significant decreases in both the spleen index and thymus index (*p* < 0.001; Supplementary Figures S1B, C). Intragastric administration of E-Jiao significantly (*p* < 0.01; Supplementary Figure S1B) increased the spleen index of the mice. Notably, the administration of LMEJ led to a dose-dependent increase in both the spleen index and thymus index. When LMEJ was administered at a dose of 1.56 g/kg, a significant difference was observed in the thymus index between the LMEJ-H and Model groups (*p* < 0.05; Supplementary Figure S1C).

### 3.4 Effects of LMEJ on the peripheral blood cells of mice

**FIGURE 3:**
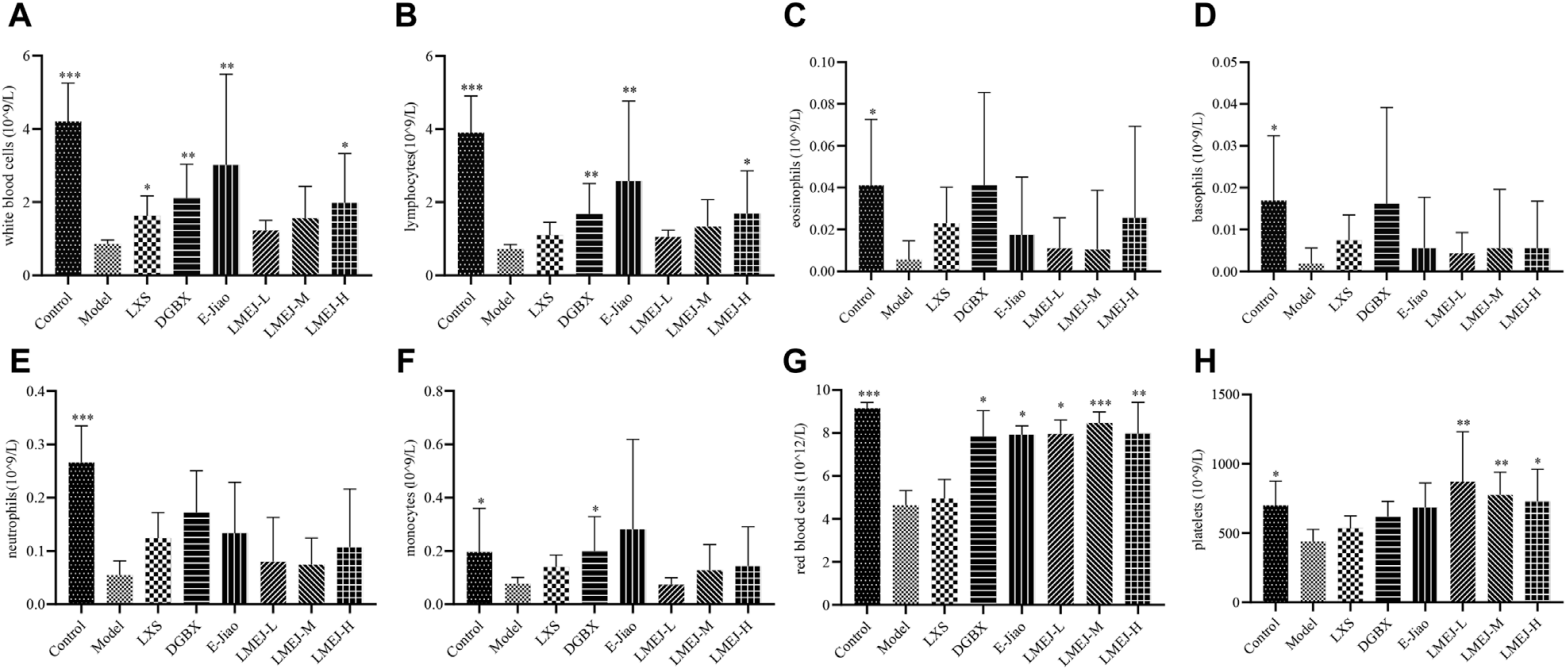
LMEJ attenuated the decrease in peripheral blood cell count induced by cyclophosphamide. **(A)** White blood cells. **(B)** lymphocytes. **(C)** eosinophils. **(D)** basophils. **(E)** neutrophils. **(F)** monocytes. **(G)** red blood cells. **(H)** platelets. The values are expressed as the mean ± SD (n = 8). *p < 0.05, **p < 0.01, ***p < 0.001 vs. the Model group.

As shown in Figure 3, the administration of cyclophosphamide led to a significant decrease (*p* < 0.001 or *p* < 0.05) in the quantity of WBCs (Figure 3A), LYs (Figure 3B), EOs (Figure 3C), BAs (Figure 3D), NEs (Figure 3E) and MOs (Figure 3F), as well as RBCs (Figure 3G) and PLTs (Figure 3H). Compared to the Model group, the administration of LXS, DGBX, E-Jiao and LMEJ-H significantly (*p* < 0.05 or *p* < 0.01) increased the WBC count (Figure 3A). The administration of DGBX, E-Jiao and LMEJ also resulted in a significant (*p* < 0.05, *p* < 0.01 or *p* < 0.001) increase in the number of RBCs (Figure 3G). Although LXS could also increase the RBC count, no significant difference was observed. Moreover, compared to those in the Model group, the PLT counts increased in all six treatment groups, but the increase was significant (*p* < 0.05 or *p* < 0.01) in only the three LMEJ groups (Figure 3H).

### 3.5 E-Jiao-derived peptides in rat blood

**FIGURE 4:**
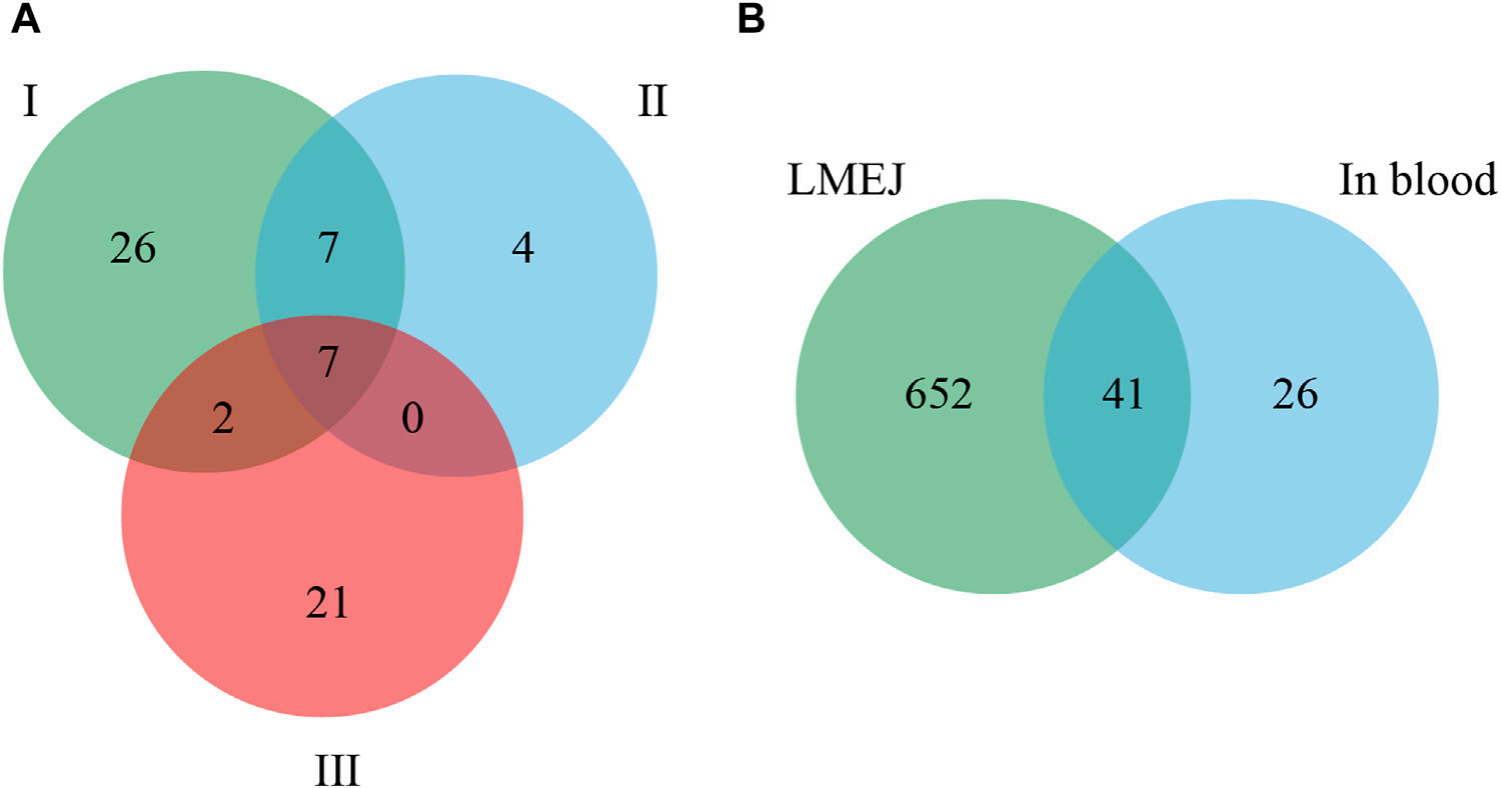
Comparison of E-Jiao-derived peptides identified in rat blood with LMEJ. **(A)** Venn diagram of E-Jiao-derived peptides identified in rat blood from triplicate experiments. **(B)** Venn diagram of peptides in rat blood and those in LMEJ.

This experiment was repeated three times. As shown in the Venn diagram (Figure 4A), 42, 18, and 30 E-Jiao-derived peptides were identified in rat blood across the replicates. Moreover, a total of 67 distinct peptides were detected in the three replicate experiments (Supplementary Table S2). Nine peptides recurred twice within the triplicate experiments, while seven peptides were consistently identified across all three replicates (Table 1).

**TABLE 1:** E-Jiao-derived peptides repeatedly identified from triplicate experiments.

Sequence	Protein	Position	AAs	MW [Da]	0 h	1 h	3 h	Score^a^
PGPAGPAGPR***	F6RTI8	271-280	10	875.46069	No^b^	Yes^c^	Yes	0.87
GPAGPSGPPGK**	F6R4Y3	1113-1123	11	920.47092	No	Yes	Yes	0.87
GPAGPQGPR**	A0A5F5Q281	1142-1150	9	835.42939	No	Yes	No	0.82
GVQGPPGPAGPR**	A0A5F5Q281	743-754	12	1088.57203	No	Yes	Yes	0.78
GRPGAPGPAGAR**	A0A5F5Q281	310-321	12	1062.56761	No	No	Yes	0.78
GVVGPQGAR***	F6RTI8	155-163	9	839.46069	No	Yes	Yes	0.30
GEAGPQGAR***	A0A5F5Q281	410-418	9	841.40357	No	Yes	Yes	0.38
DGEAGAQGPPGPAGPAGER***	A0A5F5Q281	670-688	19	1689.77001	No	Yes	Yes	0.72
GDAGPAGPK***	A0A5F5Q281	277-285	9	768.37595	No	Yes	Yes	0.63
GETGEQGDR***	A0A5F5Q281	1154-1162	9	947.39379	No	No	Yes	0.08
GPPGSAGAPGK***	A0A5F5Q281	1199-1209	11	894.45527	No	Yes	Yes	0.72
GASGPAGVR**	F6RTI8	422-430	9	770.40284	No	Yes	Yes	0.57
VGAPGPAGAR**	F6RTI8	223-232	10	851.46069	No	Yes	Yes	0.58
PGLPGPSGEPGK**	A0A5F5Q281	1030-1041	12	1091.56046	No	Yes	No	0.70
GADGSPGKDGVR**	A0A5F5Q281	809-820	12	1114.53604	No	Yes	Yes	0.48
ISVPGPMGPSGPR**	A0A5F5Q281	174-186	13	1250.64348	No	Yes	Yes	0.72

**and *** indicate that the peptide was identified two and three times from triplicate experiments, respectively.

^a^ Represents the scores calculated by PeptideRanker.

^b^ Indicates that the peptide was not detected in the corresponding samples.

^c^ Indicates that the peptide was detected in the corresponding sample.

Among all the identified peptides, 65, accounting for 97% of the total peptides, were derived from type I collagen, and the remaining two peptides were released from type III collagen (Supplementary Table S2). The lengths of the 67 E-Jiao-derived peptides ranged from 7 to 19 amino acids, while their molecular weights ranged from 700.33198 to 1689.77001 Da. Moreover, the presence of these peptides was affected by the time elapsed after administration. For example, the peptide GVVGPQGAR was detectable at both 1-h and 3-h post-ingestion, while the peptide GETGEQGDR was exclusively detected at 3 h post-ingestion. In the case of GPAGPQGPR, it was present at 1-h post-ingestion but absent at 3-h post-ingestion (Table 1; Supplementary Table S2). Among the 67 peptides, 41 were also found in LMEJ (Figure 4B; Supplementary Table S2), while the remaining 26 peptides were exclusively detected in blood samples. Five peptides, including PGPAGPAGPR (PP-1), GPAGPSGPPGK (PP-2), GPAGPQGPR (PP-3), GVQGPPGPAGPR (PP-4) and GRPGAPGPAGAR (PP-5) (Table 1) were selected for synthesis.

### 3.6 Blood-supplementing effect of the E-Jiao-derived peptides

**FIGURE 5:**
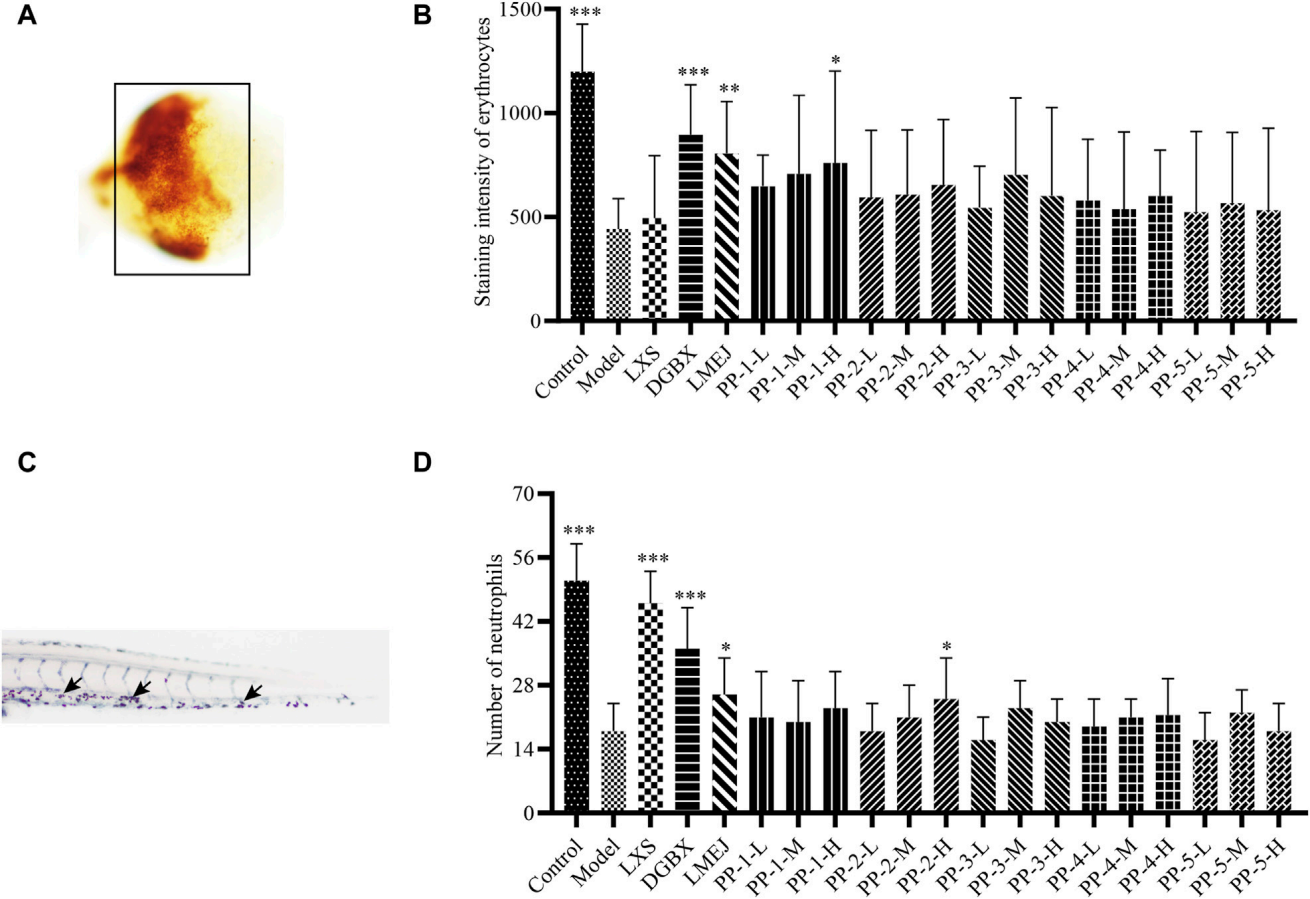
Effect of E-Jiao-derived peptides on erythrocytes in doxorubicin-induced myelosuppressed zebrafish and neutrophils in vinorelbine-induced myelosuppressed zebrafish. **(A)** Images of erythrocytes from zebrafish. **(B)** PP-1 protects zebrafish from the doxorubicin-induced decrease in erythrocyte counts. **(C)** Images of neutrophils from zebrafish stained with Sudan Black B.** (D)** PP-2 alleviated the vinorelbine-induced reduction in neutrophil count in zebrafish. Erythrocytes are indicated by a black box, and neutrophils are indicated by black arrows. The values are expressed as the mean ± SD (n ≥ 60). *p < 0.05, **p < 0.01, ***p < 0.001 vs. the Model group.

As shown in Figure 5, among the five selected E-Jiao-derived peptides, PP-1 and PP-2 exhibited protective effects against myelosuppression in zebrafish. PP-1 significantly (*p* < 0.05) increased the number of zebrafish erythrocytes at a concentration of 40 μg/mL. Additionally, PP-2 significantly (*p* < 0.05) increased the number of neutrophils in zebrafish at concentrations of 15 μg/mL.

### 3.7 Common targets and key targets of peptide-disease

**FIGURE 6:**
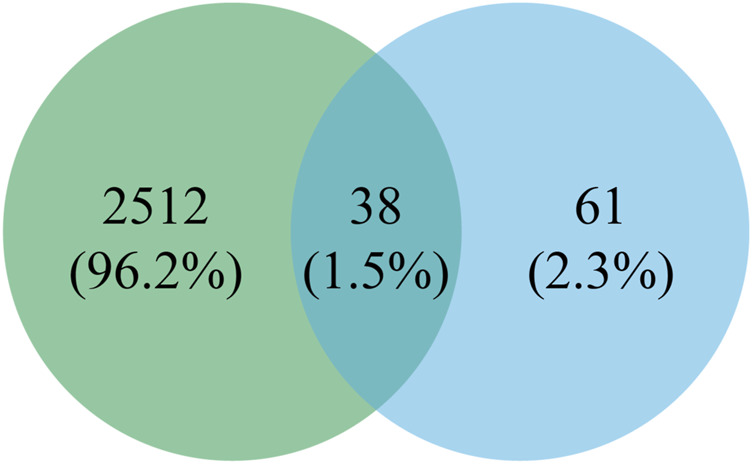
Venn diagram of peptides and disease intersection targets.

**FIGURE 7:**
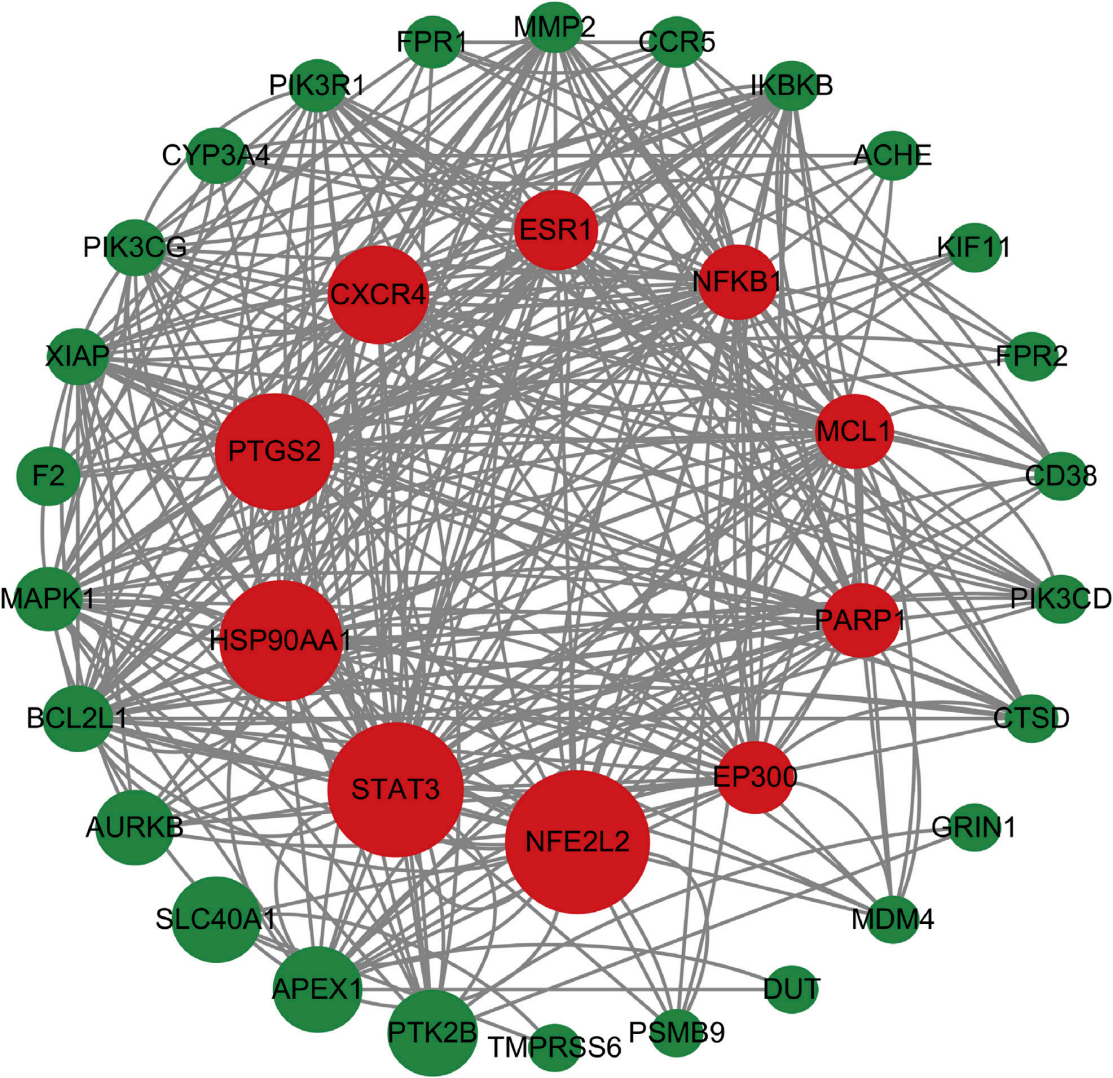
The PPI network. The size of the nodes in the network corresponds to the betweenness centrality of the targets. Nodes in red color represent the key targets.

As shown in Figure 6, a total of 2550 targets associated with blood deficiency were identified by searching the GeneCards and OMIM-GENE-MAP databases. PP-1 and PP-2 were retrieved from the Super PRED database, leading to the identification of 99 potential targets. A Venn diagram was used to find the intersection of drug targets and disease targets, resulting in 38 common targets. These 38 gene targets were inputted into the STRING database and visualized using Cytoscape 3.10.1 (Figure 7). The size of the nodes in the network corresponds to the betweenness centrality of the targets. Using cut-off values for betweenness centrality (37.0000), closeness centrality (0.0146), and degree centrality (19.1111), 10 key targets, including NFE2L2, STAT3, HSP90AA1, and others were screened.

### 3.8 GO and KEGG enrichment analysis of key targets

**FIGURE 8:**
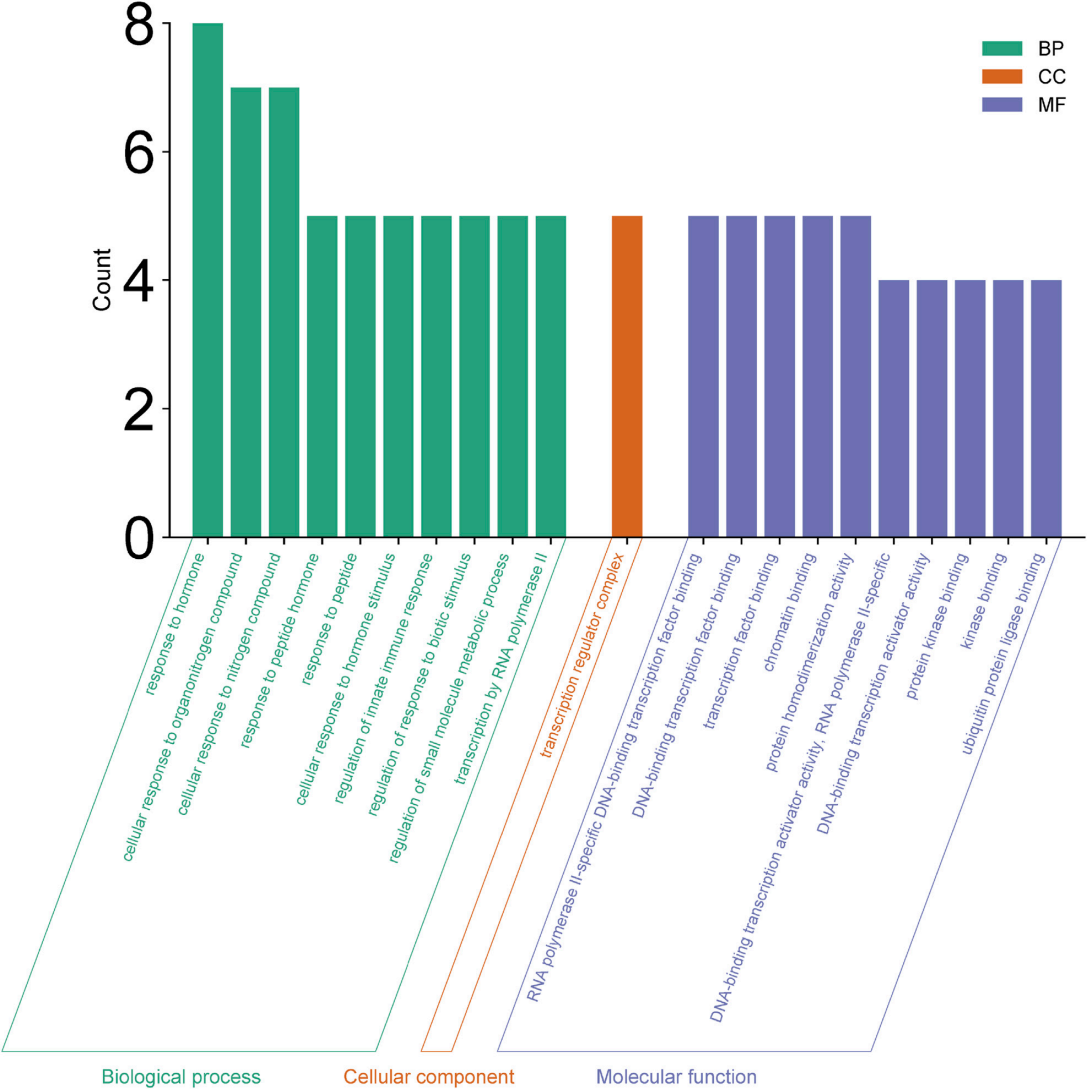
GO enrichment analysis.

**FIGURE 9:**
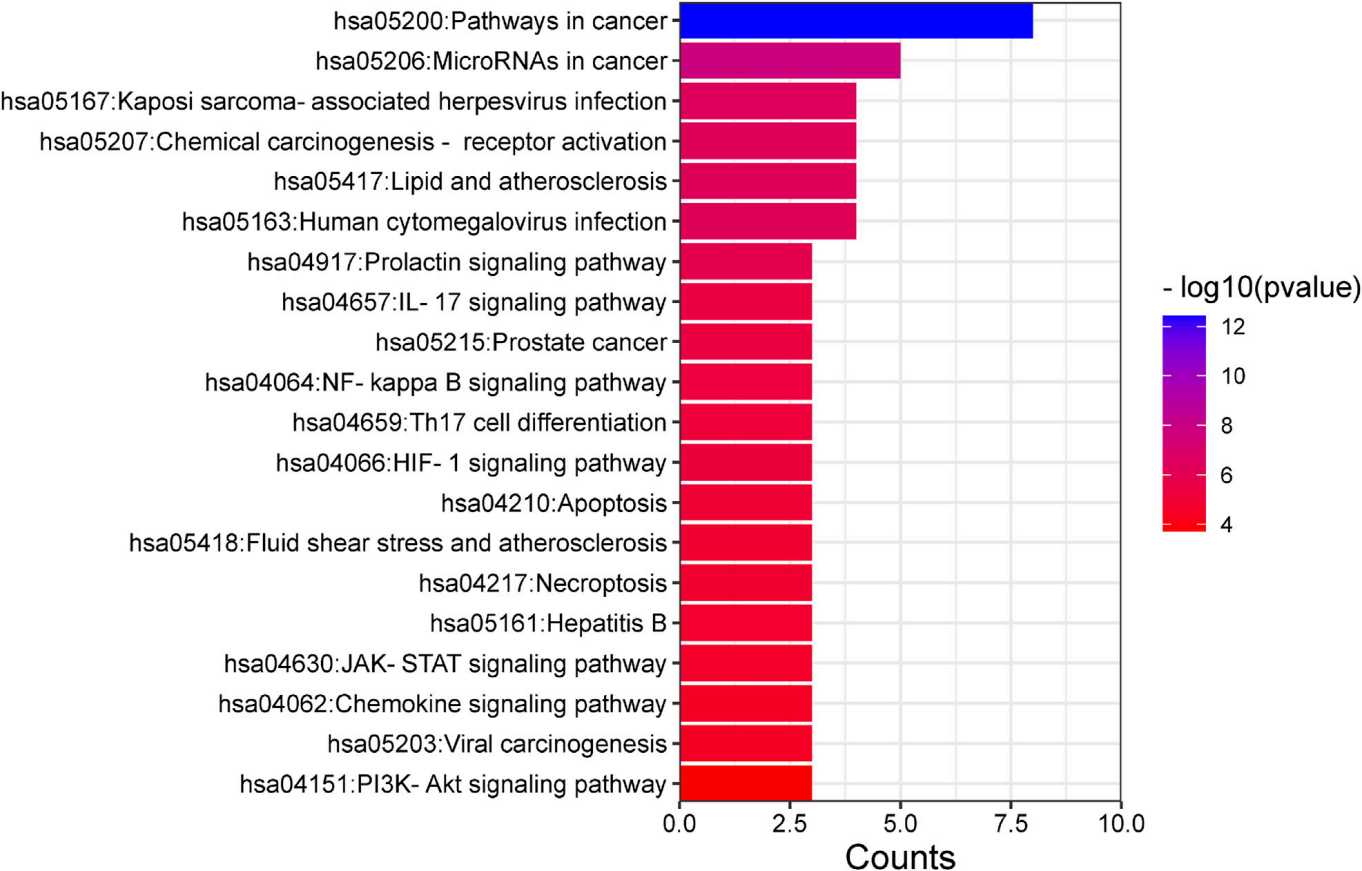
KEGG pathway enrichment analysis.

Enrichment analyses for GO and KEGG pathways were conducted on 10 key targets using the Metascape database (*p* < 0.05). The GO enrichment analysis yielded a total of 123 records, with the biological process (108), molecular function (14), and cell composition (1) accounting for 87.80%, 11.38%, and 0.08% respectively. In the biological process category (Figure 8), the target proteins were primarily associated with the response to stimulus, the biological process involved in interspecies interaction between organisms and the metabolic process. In the molecular functions category (Figure 8), the target proteins were predominantly involved in kinase binding, transcription factor binding, chromatin binding, and transcription activation. In the cell components category (Figure 8), the target proteins were mainly part of the transcription regulator complex. The KEGG analysis identified 21 signaling pathways, including lipid and atherosclerosis, prolactin signaling pathway, IL-17 signaling pathway, HIF-1 signaling pathway, JAK-STAT signaling pathway, PI3K-Akt signaling pathway, among others (Figure 9).

### 3.9 Molecular docking analysis

**FIGURE 10:**
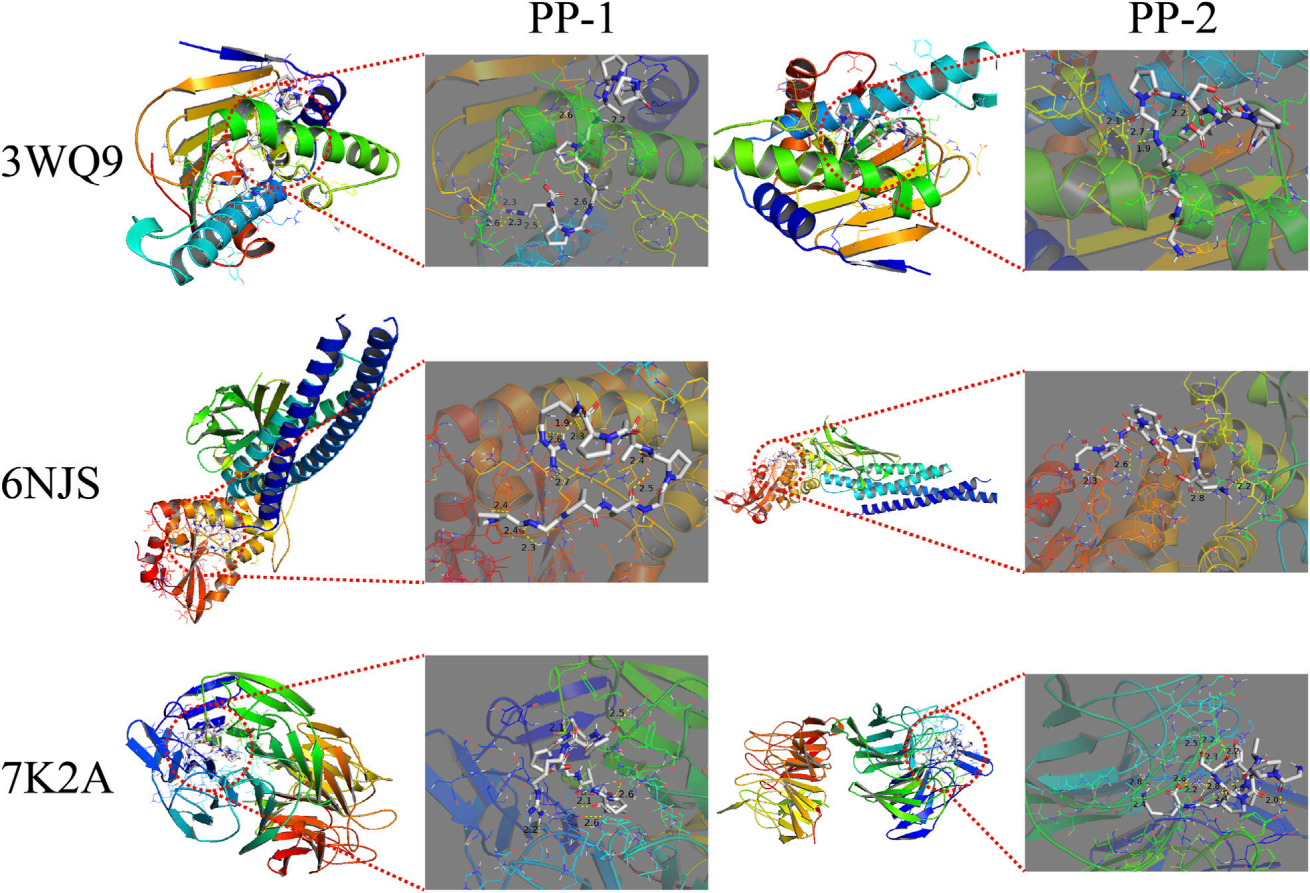
The docking results of PP-1 and PP-2 with three core targets. The peptides are displayed in STICKS mode. The yellow dotted lines indicate the hydrogen bonds formed between the peptide and the amino acid residues.

The peptides PP-1 and PP-2 were docked with the three core targets (NFE2L2, STAT3, and HSP90AA1) that had the highest betweenness centrality. The docking energy of the two peptides with the three core targets was found to be less than −5.5 kcal/mol (Table 2). Furthermore, PP-1 and PP-2 formed multiple hydrogen bonds (within a 4 Å radius) with the amino acid residues in the active center of the target proteins (Figure 10). These findings suggest that PP-1 and PP-2 exhibit strong binding activity with the three core targets.

**TABLE 2:** Docking affinity and formed hydrogen bonds of peptides (PP-1 and PP-2) to core targets.

Peptides	Gene	PDB ID	Affinity (kcal/mol)	Hydrogen bonds (≤4 Å)
PP-1 (PGPAGPAGPR)	NFE2L2	7K2A	−10.0	6
	STAT3	6NJS	−7.1	9
	HSP90AA1	3WQ9	−10.6	7
PP-2 (GPAGPSGPPGK)	NFE2L2	7K2A	−8.4	12
	STAT3	6NJS	−6.6	4
	HSP90AA1	3WQ9	−8.5	4

## 4 Discussion

E-Jiao, a protein-based traditional Chinese medicine, has been widely used to treat myelosuppression induced by chemotherapy or radiotherapy. However, the specific components responsible for the blood-supplementing effect of E-Jiao have not been identified. In the present study, we tested the hypothesis that low molecular weight peptides are responsible for E-Jiao’s effect. We prepared LMEJ using an *in vitro* digestion method and subsequently characterized it using a peptidomics method. More than half of the peptides within LMEJ were derived from collagens, especially type I collagen. The blood-supplementing effect of LMEJ was assessed using a myelosuppressed zebrafish model and a murine model. The results showed that LMEJ exhibited a blood-supplementing effect in both zebrafish and mice. Analysis of rat blood samples revealed that most of the peptides entering the bloodstream were derived from type I collagen. Two novel E-Jiao-derived peptides, identified in rat blood, were found to alleviate myelosuppression in zebrafish. The mechanism underlying the blood-supplementing effect of E-Jiao was also investigated by network pharmacology. These results suggest that LMEJ is responsible for the blood-supplementing effect of E-Jiao. This study provides new insights into the effective components of E-Jiao.

Characterization of LMEJ led to the identification of a total of 693 peptides, most of which originated from collagen proteins. This result is consistent with previous studies that have shown that collagens, especially type I collagen, are the main components of E-Jiao (Li et al., 2017; Zhang et al., 2022). Previous studies have shown that doxorubicin and vinorelbine can cause hematological toxicity, leading to reductions in RBC and WBC counts (Chen et al., 2017; Li et al., 2020). In the present study, a significant reduction in the count of RBCs and neutrophils was observed in zebrafish following treatment with doxorubicin and vinorelbine. Danggui Buxue Koufuye is a well-known decoction that is commonly used to treat various types of blood-deficient syndromes (Shi et al., 2020). Leucogen is a cysteine derivative that is clinically used to treat radiation- or chemotherapy-induced leukopenia by increasing WBC counts (Huang et al., 2014). Notably, treatment with LMEJ reversed the effects of doxorubicin and vinorelbine, similar to DGBX and LXS. This suggests that LMEJ has a protective effect on chemotherapy-induced myelosuppression in zebrafish. Interestingly, this protective effect was not observed in zebrafish treated with E-Jiao. Treatment with cyclophosphamide led to significant reductions in various peripheral blood cell counts, including RBCs and WBCs. The administration of LXS could alleviate the toxicity caused by cyclophosphamide and significantly increase the quantity of WBCs, which was consistent with the findings of a previous study (Huang et al., 2014). DGBX could elevate both the WBC and RBC counts in myelosuppressed mice. Notably, treatment with LMEJ also successfully reversed the cyclophosphamide-induced reduction in blood cell counts, body weight and thymus visceral index, indicating that LMEJ had a protective effect on cyclophosphamide-induced myelosuppression in mice. Interestingly, E-Jiao exhibited efficacy comparable to that of LXS and DGBX, indicating that its blood-supplementing effect was similar to that observed in previous studies (Tian et al., 2017; Zhang et al., 2019). It has been demonstrated that hematopoiesis in zebrafish begins at 24 hpf, while the digestive system of zebrafish is not fully functional until 5 days post-fertilization (Ng et al., 2005; Kulkeaw and Sugiyama, 2012). Therefore, the zebrafish were unable to digest the protein-based E-Jiao at 48 hpf. However, E-Jiao was digested and absorbed when administered to mice. This may explain why LMEJ was able to provide a blood-supplementing effect in both zebrafish and mice, whereas E-Jiao was only able to exert a protective effect in mice. These findings suggested that E-Jiao may have to be digested into peptides to exert its blood-supplementing effect.

It is believed that peptides must reach the bloodstream to exert their activities (Miner-Williams et al., 2014). In this study, a total of 67 E-Jiao-derived peptides were identified in rat blood samples. Notably, only seven of these peptides were consistently identified across the three replicate experiments, indicating relatively large variability. Several previous studies have reported similar findings (Picariello et al., 2019; Zhu et al., 2019; Sheng et al., 2021). This variability could arise from several factors, such as variable digestion and absorption capacities, the influence of other dietary components and the possibility of the loss of random peptides during sample preparation (Dingess et al., 2019; Caira et al., 2022). The process of collecting rat blood samples for peptide identification spanned approximately 3 weeks. The intestinal digestion and nutrient absorption capability of the rats may have changed during this period. Additionally, rat chow residue was observed in the stomach and intestine, despite the rats being fasted for 24 h before E-Jiao administration. The coconsumption of other foods could increase the complexity of biological matrices and alter intestinal permeability, potentially significantly influencing on the absorption and detection of E-Jiao-derived substances.

Among the 67 E-Jiao-derived peptides identified in rat blood, 97% (65 peptides) were released by type I collagen. This may be attributed to the high abundance of type I collagen in donkey skin, which is in good agreement with the LMEJ characterization data. Notably, the lengths of these 67 peptides ranged from 7 to 19 amino acids. It has been demonstrated that peptide length is closely correlated with the pathway by which the peptide is transported across the intestinal epithelium. For instance, peptides longer than three amino acids often traverse via paracellular transport through tight junctions or transcytosis via vesicles (Xu et al., 2019). Such variation in the transport pathway influences the absorption and distribution of exogenous peptides. Previous studies have indicated that exogenous peptides in plasma can persist for several minutes to several hours following the ingestion of specific peptide- or protein-enriched substances (Taga et al., 2016; Hanh et al., 2017; Nwachukwu et al., 2019). In this study, the detection of certain peptides was time-dependent, suggesting that their absorption into and subsequent clearance from the bloodstream occurred at different rates. Notably, approximately 70% (47) of the total peptides were detected 1 h after E-Jiao administration. These findings suggest that these peptides may be absorbed more rapidly into the bloodstream than those not detected at this time point (Supplementary Table S2). These findings differ slightly from earlier studies on dipeptides, tripeptides and tetrapeptides, where peptides absorbed into the bloodstream were detected within 1 h (Taga et al., 2016; Hanh et al., 2017; Nwachukwu et al., 2019). However, further studies are needed to explore whether these differences could be attributed to the lengths of the peptides.

After entering the bloodstream, peptides may be further degraded by carboxypeptidases and aminopeptidases (Sato, 2017). In the present study, certain peptides with similar amino acid sequences were detected in the blood. For example, a nonapeptide, GPIGPVGAR, derived from regions 1133-1141 of the type I collagen alpha 1 chain, was identified in blood samples. Interestingly, other peptides originating from the same region, such as AGPIGPVGARGPAGP, AGPIGPVGARGP and GPIGPVGARGP, were also detected. These peptides differ in length from GPIGPVGAR by several amino acids, either at the N-terminus or C-terminus. It appears that GPIGPVGAR was produced by the continuous hydrolysis of AGPIGPVGARGPAGP at both its N-terminus and C-terminus. Furthermore, a conventional *in vitro* activity-guided fractionation assay identified a potentially active peptide, VPGPMGPSGPR, which exhibited a hematopoietic stimulating effect (Wu et al., 2016). Interestingly, a closely related peptide, ISVPGPMGPSGPR, differing by only two amino acids at the N-terminus, was detected in the blood in this study. However, further research is necessary to determine whether the peptide ISVPGPMGPSGPR undergoes further degradation in the bloodstream.

Two of the peptides that entered the rat bloodstream were demonstrated to protect zebrafish from chemotherapy-induced myelosuppression by increasing red blood cell or neutrophil counts. These interesting results further demonstrate that peptides are responsible for the blood-supplementing effect of E-Jiao. Network pharmacology was employed to investigate the mechanisms through which PP-1 and PP-2 exert blood-supplementing effect. Key targets such as NFE2L2, STAT3, and HSP90AA1 were identified. The NFE2L2 gene encodes Nrf2, which primarily regulates antioxidant responses and is crucial for preventing and correcting cellular redox imbalances. Nrf2 can activate the ARE (antioxidant responsive element) to further regulate inflammation and apoptosis. The Nrf2/ARE signaling pathway serves as a vital defense mechanism against endogenous oxidative stress from various physical and chemical sources. Nrf2 has been identified as a key target for reducing damage from radiotherapy and chemotherapy (Bartolini et al., 2019; Shimura et al., 2019). STAT3 controls a variety of vertebrate functions, including immune regulation and inflammation. Activation of the STAT3 pathway enhances the survival of hematopoietic stem cells and plays a significant role in repairing hematopoietic injury (Hillmer et al., 2016; Amaya et al., 2022). Molecular docking results demonstrated that the peptide had a high affinity for all these targets, suggesting that the peptide may exert its tonic effect by modulating these targets. Moreover, GO and KEGG enrichment analysis results indicated that these PP-1 and PP-2 might perform a variety of molecular functions by regulating multiple biological processes to achieve the blood-supplementing effect. However, additional research is required to explore the detailed mechanisms through which these peptides exert their blood-supplementing effect.

In summary, we prepared LMEJ using an *in vitro* method, and peptide profiling revealed that the majority of the peptides within LMEJ were derived from collagens, especially type I collagen. LMEJ exerts a blood-supplementing effect in both zebrafish and mice, whereas E-Jiao functions only in mice. After administrating E-Jiao, a total of 67 E-Jiao-derived peptides were detected in the blood of the rats, 97% of which were derived from type I collagen. Two novel E-Jiao-derived peptides, detected in the blood of the rats, were found to protect the zebrafish from myelosuppression similar to LMEJ. Network pharmacology studies indicated that the peptides may influence targets like STAT3. This, in turn, could modulate signaling pathways, including the JAK-STAT pathway, thereby exerting a blood-supplementing effect. This study provides new insights into the effective components of E-Jiao. Further studies are needed to better understand the relationship between peptides, especially those in rat blood, and the blood-supplementing effect of E-Jiao.

## Data Availability

The data presented in the study are deposited to the ProteomeXchange Consortium (https://proteomecentral.proteomexchange.org) via the iProX partner repository with the dataset identifier PXD052456/ Supplementary material. Further inquiries can be directed to the corresponding authors.

## Supplementary material

SUPPLEMENTARY FIGURE S1 Changes in body weight and visceral indices of the mice. **(A)** Body weight changes. **(B)** Spleen visceral index changes. **(C)** Thymus visceral index changes. The values are expressed as the mean ± SD (*n* = 8). * *P* < 0.05, ** *P* < 0.01, *** *P* < 0.001 *vs.* the Model group.
