# Supplementary figures and images for: Blood-supplementing effect of low molecular weight peptides of E-Jiao on chemotherapy-induced myelosuppression: evaluation of pharmacological activity and identification of bioactive peptides released in vivo
Source: Front Pharmacol. 2024 Jun 5;15:1366407. doi: 10.3389/fphar.2024.1366407 (PMC11188354; doi:10.3389/fphar.2024.1366407)

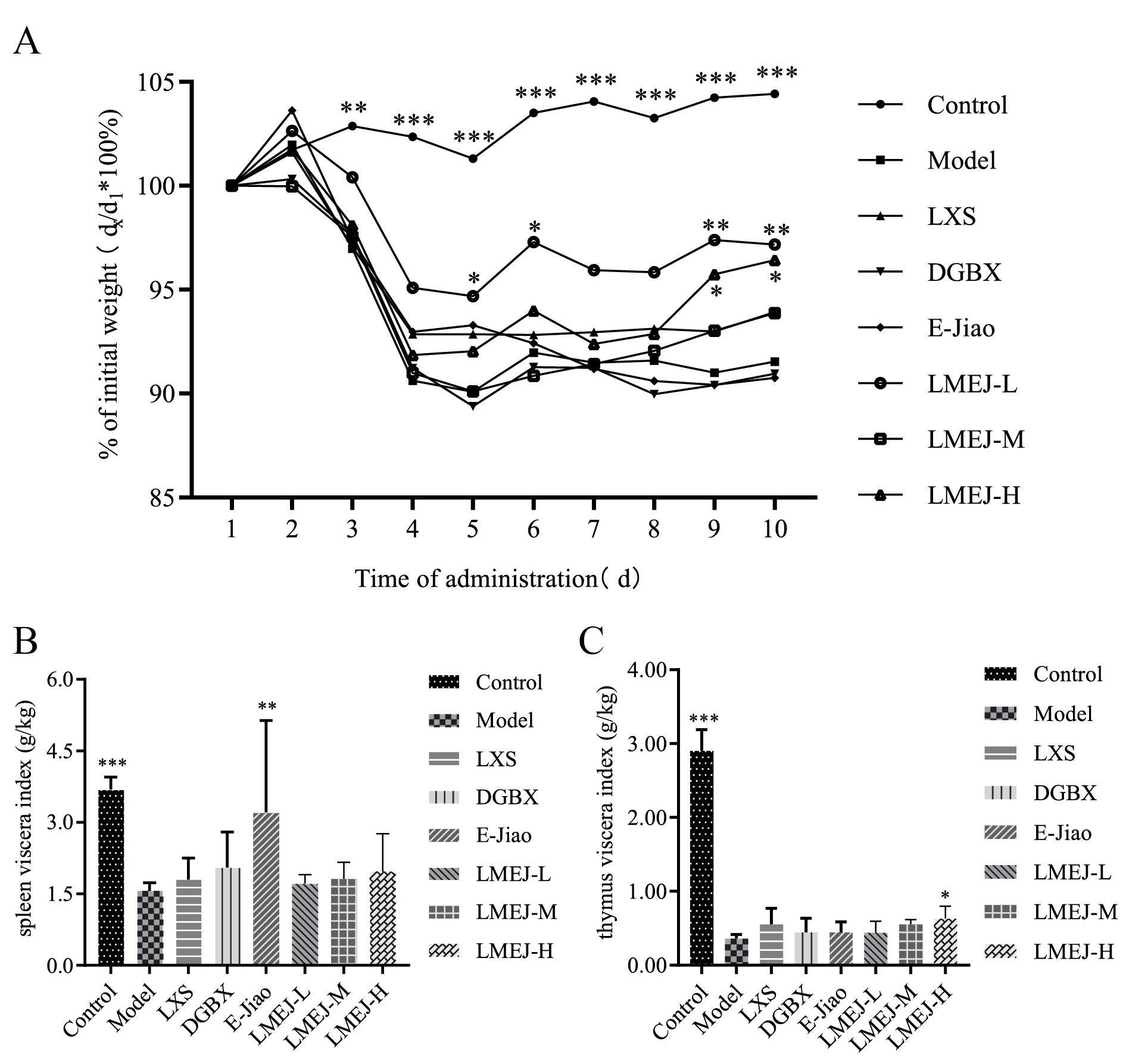

Supplement: Supplementary file 2 [file Image1.TIF]
